# The Hippo Pathway Promotes Notch Signaling in Regulation of Cell Differentiation, Proliferation, and Oocyte Polarity

**DOI:** 10.1371/journal.pone.0001761

**Published:** 2008-03-12

**Authors:** Jianzhong Yu, John Poulton, Yi-Chun Huang, Wu-Min Deng

**Affiliations:** Department of Biological Science, Florida State University, Tallahassee, Florida, United States of America; Centre National de la Recherche Scientifique, France

## Abstract

Specification of the anterior-posterior axis in *Drosophila* oocytes requires proper communication between the germ-line cells and the somatically derived follicular epithelial cells. Multiple signaling pathways, including Notch, contribute to oocyte polarity formation by controlling the temporal and spatial pattern of follicle cell differentiation and proliferation. Here we show that the newly identified Hippo tumor-suppressor pathway plays a crucial role in the posterior follicle cells in the regulation of oocyte polarity. Disruption of the Hippo pathway, including major components Hippo, Salvador, and Warts, results in aberrant follicle-cell differentiation and proliferation and dramatic disruption of the oocyte anterior-posterior axis. These phenotypes are related to defective Notch signaling in follicle cells, because misexpression of a constitutively active form of Notch alleviates the oocyte polarity defects. We also find that follicle cells defective in Hippo signaling accumulate the Notch receptor and display defects in endocytosis markers. Our findings suggest that the interaction between Hippo and classic developmental pathways such as Notch is critical to spatial and temporal regulation of differentiation and proliferation and is essential for development of the body axes in *Drosophila*.

## Introduction

During the development of multi-cellular organisms, a limited number of signal-transduction pathways collaborate to provide precise control of various aspects of cellular behavior. The recently identified Hippo (Hpo) tumor-suppressor pathway plays an important role in restricting organ size by regulation of proliferation and apoptosis; reviewed by [1], [2], [3], [4], [5]. The hierarchy of components of the pathway identified so far includes two FERM (4.1, Ezrin, Radixin, Moesin) domain–containing proteins, Merlin (Mer) and Expanded (Ex); the Ste20 family kinase Hpo and its cofactor Salvador (Sav); the nuclear Dbf2–related (NDR) family kinase Warts (Wts, also known as Lats) and its cofactor Mats (Mob as a tumor suppressor); and a transcriptional coactivator Yorkie (Yki) [6], [7], [8], [9], [10], [11], [12], [13], [14], [15], [16]. In addition, several studies identified the atypical cadherin Fat (Ft) as a potential receptor upstream of Ex in the Hpo pathway, indicating that this pathway might be regulated by extracellular signals [17], [18], [19]. Another study, however, suggested that Ft regulates the Wts protein level directly [20]. Known targets of the Hpo pathway include the cell survival and proliferation regulators *cyclin E* (*cycE*) and *diap1* and a microRNA, *bantam* [21], [22]. *ex* and *mer* are also downstream targets of the pathway in a feedback loop [15]. Although most studies focus on revealing novel components of this pathway and its role in cell proliferation and growth control, the interaction with other signal-transduction pathways in the regulation of cellular processes remains largely unexplored.

An excellent model system for investigating how multiple signaling pathways interact to regulate cell differentiation and proliferation is the *Drosophila* follicle-cell epithelium (FE), which surrounds the germ-line cells to form an egg chamber [23]. The development of the egg chamber, referred to as oogenesis, can be divided into 14 morphologically distinct stages; reviewed in [24]. During stages 6/7 of oogenesis, communication between the germ-line cells and the somatically derived follicle cells induces two major changes in the FE that are important for proper progression of oogenesis. First, the follicle cells stop the normal mitotic cycle, differentiate, and enter three rounds of the endoreplication cycle (also called the endocycle). Second, the follicle cells at the posterior end of the egg chamber are induced to take the posterior follicle cell (PFC) fate, whereas the anterior follicle cells adopt a “default” fate and express anterior cell fate markers.

Three well-characterized signaling pathways have been shown to be involved in the temporal and spatial regulation of follicle-cell differentiation and proliferation during this stage of oogenesis: the Notch pathway, which is activated by the germ-line-expressed ligand Delta [25], [26], [27]; the epidermal growth factor receptor (EGFR) pathway, which is activated by secretion of a transforming growth factor α (TGFα) homolog, Gurken (Grk), from the oocyte [28], [29]; and the Janus kinase-signal transducer and activator of transcription (JAK-STAT) signaling pathway, which is activated by an Unpaired (Upd) gradient produced by the polar follicle cells located at the anterior and posterior ends of the egg chamber [30]. Notch signaling controls the temporal pattern of follicle-cell differentiation and proliferation, as it induces the switch from the mitotic cycle to the endocycle in follicle cells, as well as a switch from an “immature” to a “mature” cell fate [26], [27]. The EGFR and JAK-STAT pathways further regulate the pattern of follicle-cell differentiation along the anterior-posterior (AP) axis of the egg chamber. The combined effect of these pathways leads to proper differentiation of the PFC, which are thought to send a signal back to the oocyte to establish the AP polarity of the oocyte. Disruption of any of these three pathways in the PFC causes AP polarity defects in the oocyte; reviewed in [31].

Here we report that the Hpo pathway regulates follicle-cell differentiation and oocyte-polarity formation through its interaction with the Notch pathway. We also provide evidence that the Hpo pathway may be required for correct endocytic trafficking in the follicle cells, including the Notch receptor itself. Our studies reveal novel crosstalk between these two important pathways in the development of the *Drosophila* egg chamber.

## Results

### The Hippo pathway is required in follicle cells for oocyte polarity formation

**Figure 1:**
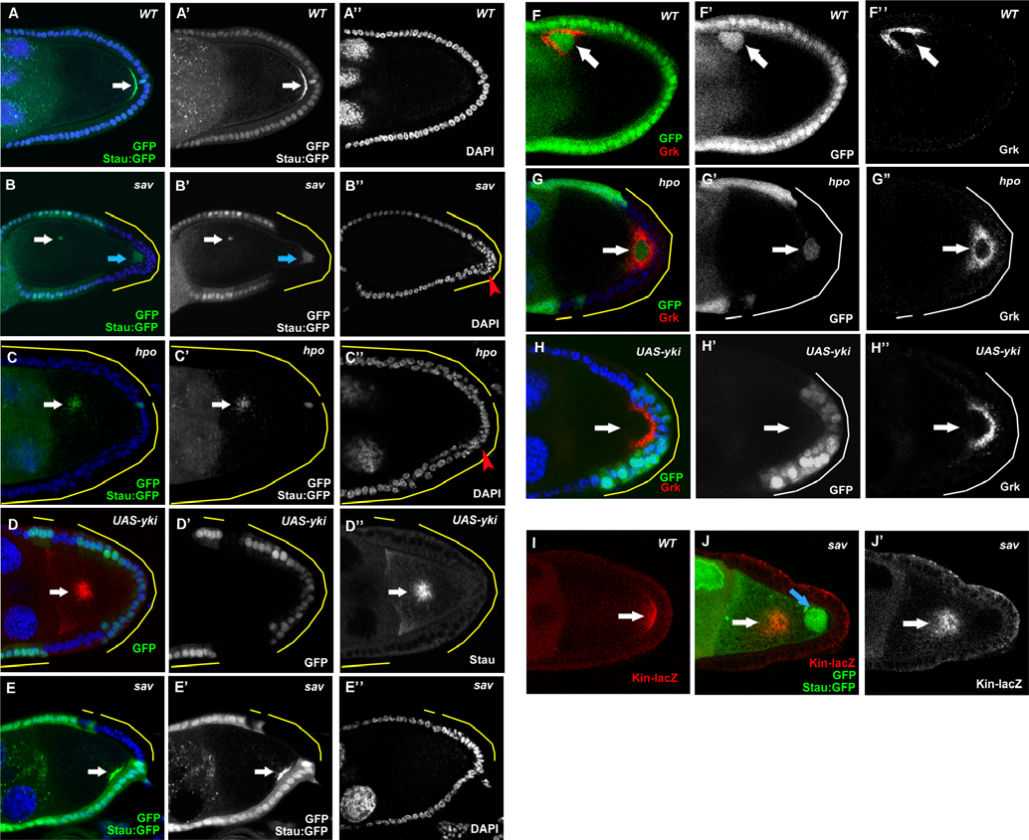
The Hpo pathway is required for oocyte polarity formation. (A) Stau:GFP (arrow) is localized to the posterior of wild-type stage-9 oocytes. (B) Large *sav* follicle-cell clones cause a complete mislocalization of Stau-GFP (white arrow) toward the oocyte center, and the oocyte nucleus (blue arrow) remains at the posterior. (C) A stage 9 egg chamber with large *hpo* PFC clone also shows mislocalization of Stau:GFP toward the center of the oocyte (arrow). (E) Stau (arrow) is mislocalized away from the region adjacent to the *sav* clones when the PFC are partially mutated. (F) Oocyte nucleus and Grk (arrow) are localized to the dorsal anterior corner of wild-type stage-9 oocytes. (G) Large *hpo* follicle-cell clones cause mislocalization of the oocyte nucleus and Grk (arrow) at the oocyte posterior. Overexpression of Yki also caused Stau (D, arrow) and Grk (H, arrow) mislocalization. (I) Plus ends of microtubules, visualized with Kin:β-Gal (arrow) localization at the posterior of a wild-type stage-9 oocyte. (J) A stage-9 egg chamber with a large *sav* follicle-cell clone showing abnormal Kin:β-Gal (arrowhead) localization in the center of the oocyte, as well as mislocalization of the oocyte nucleus (blue arrow). Multilayering and small nuclear phenotypes can be observed in PFC clones of both *sav* and *hpo* mutants (red arrowheads). Loss-of-function clones are marked as the GFP-negative cells. Gain-of-function clones (*UAS-Yki*) are GFP-positive. All clones are additionally highlighted by yellow lines to indicate the affected follicle cells, except in a few cases of complete or almost complete follicle cell clones. In all Figures, posterior is to the right. Nuclei are marked in most figures by DAPI staining in blue.

**Figure 2:**
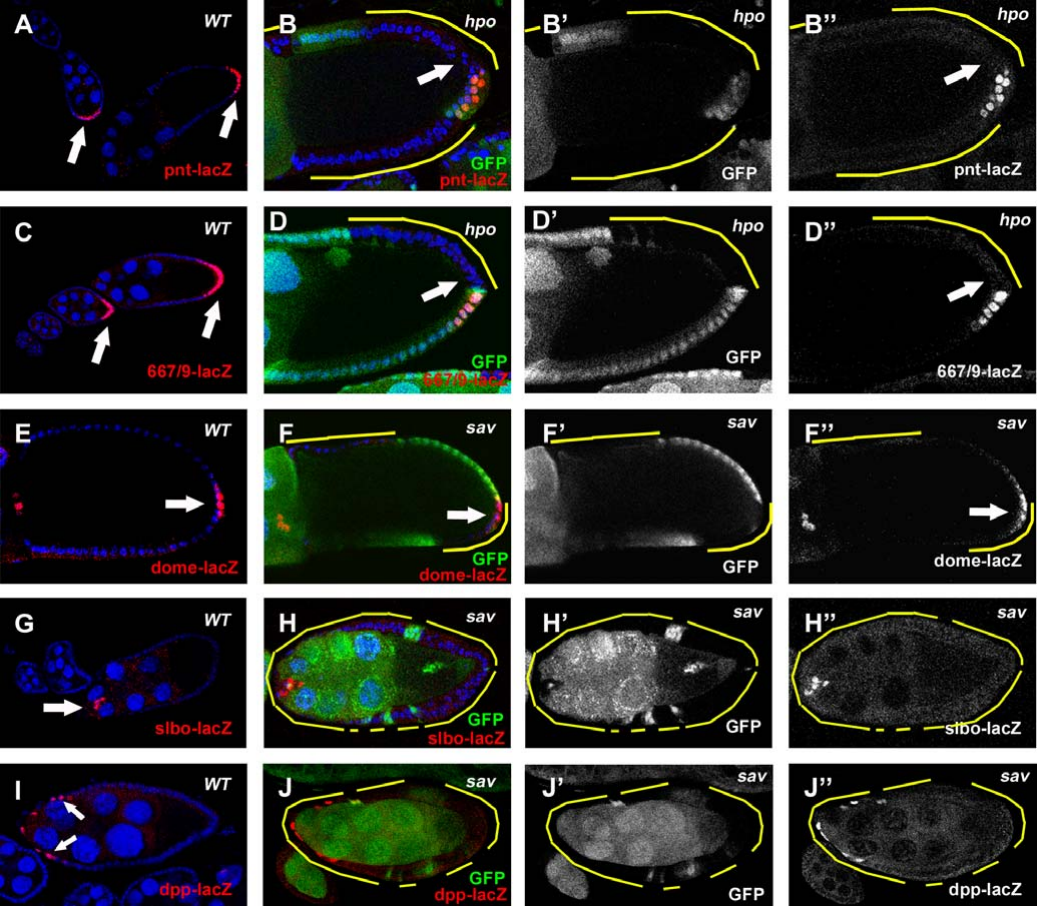
Mutants of the Hpo pathway disrupt PFC differentiation. The PFC markers *pnt-lacZ* (A) and *667/9-lacZ* (C) are specifically expressed in the PFC after stage 6 in wild-type egg chambers. (B and D) *hpo* PFC clones fail to express *pnt-lacZ* (B) or *667/9-lacZ* (D), in a cell-autonomous manner (arrows). (E) Activation of JAK-STAT signaling in the PFC (arrow) can be marked by the expression of *dome-lacZ*. (F) In *sav* PFC clones, expression of *dome-lacZ* is not affected (arrow). The AFC markers *slbo-lacZ* (G) and *dpp-lacZ* (I) are expressed in the AFCs in stage-9 wild-type egg chambers. In *sav* mutant PFC, no misexpression of *slbo-lacZ* (H) or *dpp-lacZ* (J) was detected.

**Figure 3:**
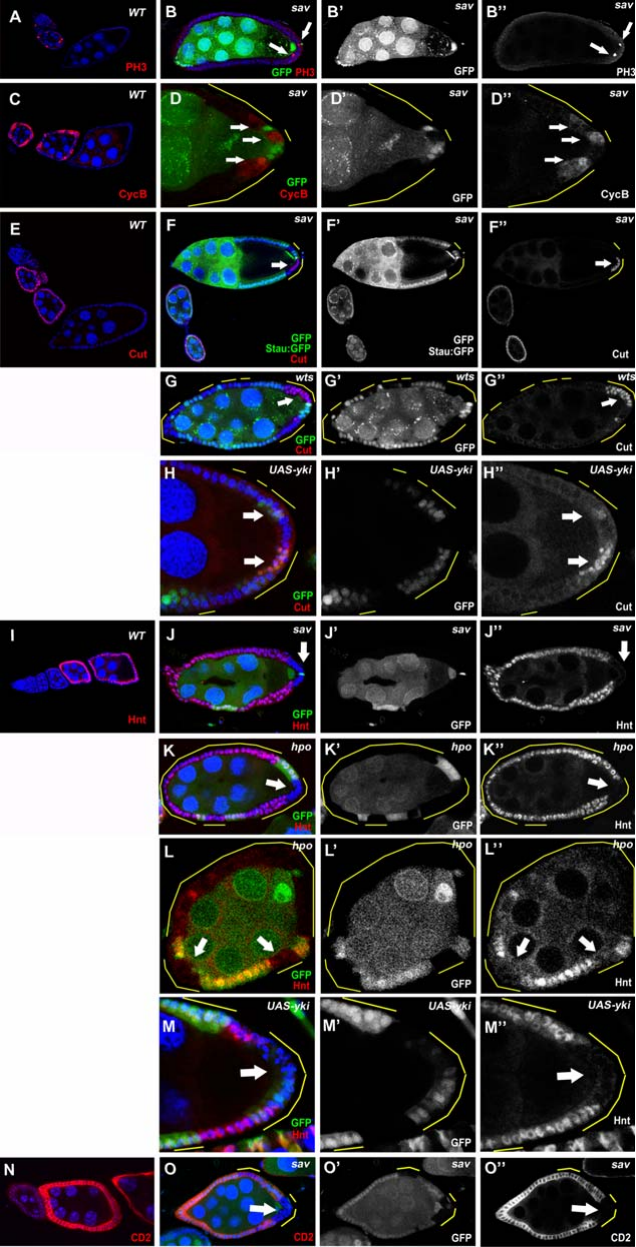
Notch signaling is disrupted in PFC clones of Hpo pathway mutants. (A and C) PH3 and Cyclin B are expressed sporadically in immature follicle cells during early stages (S1–S6) in wild-type egg chambers. In *sav* mutants, staining of PH3 (B) and Cyclin B (D) was occasionally found in mutant PFC after stage 6 (arrows). (E) In wild-type egg chambers, Cut is expressed in follicle cells until about stage 6. (F and G) Prolonged Cut expression was found in *sav* (F) and *wts* (G) PFC clones at stages 8–10 of oogenesis. (I) Hnt is expressed in follicle cells after stage 6 in the wild type. No Hnt expression was found in *sav* (J) or *hpo* (K) mutant PFC in stage-8 egg chambers. (L) Lack of Hnt staining was also occasionally observed in anterior and lateral hpo clones in stage 7 egg chambers (arrows). Overexpression of Yki caused prolonged Cut expression (H) and decreased Hnt expression (M). (N) The E(spl):CD2 Notch activity reporter, visualized by CD2 staining, is upregulated in follicle cells during stages 7–10A of oogenesis in wild-type egg chambers. (O) Lack of CD2 staining was observed in *sav* mutant PFC in this stage-7 egg chamber (arrow).

To investigate the role of the Hpo pathway in oogenesis, we generated clones mutant for three core components of the pathway (*sav*, *hpo,* and *wts*). Germ-line clones of the null-allele mutations, *sav^shrp^* [6], *hpo^42–47^* [12], and *wts^x1^* [32], did not display any obvious defects during oogenesis, suggesting that the Hpo pathway is dispensable for germ-line development (data not shown). In contrast, when large follicle-cell clones of the *sav*, *hpo*, or *wts* mutations covered the posterior end of the oocyte, we observed multilayering of the follicular epithelium with smaller nuclear size (Fig. 1B,C, 3G), as well as strong oocyte-nucleus-positioning defects (Fig. 1B,G,J, 2H, 3B,J), similar to recent studies of Hpo signaling in oogenesis [33], [34]. Normally, the oocyte nucleus migrates from a posterior location to the dorsal-anterior corner at stage 7 and stays there for the remainder of oogenesis (Fig. 1F). In mosaic egg chambers possessing large PFC clones of *hpo* or *sav* mutations, oocyte nuclei failed to migrate and remained at the posterior after stage 7 (95% in *hpo*, n = 63; 93% in *sav*, n = 76) (Fig. 1G,J). This phenotype was confirmed by staining of Grk, which is localized in close proximity to the oocyte nucleus during oogenesis (Fig. 1F,G). Because mislocalization of the oocyte nucleus and Grk indicates oocyte polarity defects, we used other oocyte polarity markers to characterize these phenotypes further. Staufen (Stau), an RNA-binding protein that colocalizes with *osk* RNA to the oocyte posterior during stages 9 and 10 of oogenesis [35](Fig. 1A), was mislocalized toward the center of the oocyte when follicle-cell clones of any of the three mutants covered the entire posterior end of the oocyte (*sav,* 97%, n = 71; *hpo,* 93%, n = 54; *wts,* 85%, n = 66; visualized by localization of Stau:GFP or Stau antibody; Fig. 1B, C and data not shown). In egg chambers only partially covered by mutant PFC clones, Stau was not present in the region of the oocyte cortex adjacent to the clones, whereas the region next to the wild-type PFC did have Stau localization (*sav,* 74%, n = 61; *wts,* 88%, n = 42) (Fig. 1E, 3F and data not shown). This phenotype is similar to the previously reported clone-adjacency mislocalization (CAM) phenotype [30], [36], [37].

The transcriptional coactivator Yki, a phosphorylation target of Wts, regulates the transcriptional control of Hpo pathway target genes [13]. Overexpression of *yki* phenocopies the *sav*, *hpo,* and *wts* loss of function phenotypes in eye imaginal discs [13]. When *yki* was overexpressed in PFC, similar oocyte polarity defects were found: Grk mislocalization (47%, n = 55) (Fig. 1H) and Stau (57%, n = 63) (Fig. 1D).

Because oocyte polarity depends on microtubule polarity, we used a microtubule plus-end marker, kinesin-β-galactosidase (Kin:β-Gal) fusion protein to further characterize the defects in oocyte polarity in Hpo defective egg chambers [38]. Indeed, Kin:β-Gal was mislocalized from its normal posterior position at the oocyte posterior (Fig. 1I), to the center of the oocyte in *sav* mosaic egg chambers, indicating a microtubule polarity defect (Fig. 1J). Together these data suggest that the Hpo pathway and its downstream target, Yki, are required in the PFC for oocyte AP polarity formation.

### The Hippo pathway is required for follicle-cell differentiation

Because establishment of oocyte polarity requires proper differentiation of the PFC, we asked whether follicle-cell differentiation is normal in Hpo-pathway mutants. First, we examined the expression of a PFC fate marker, *pointed-lacZ* (*pnt-lacZ*), in follicle-cell clones with defective Hpo signaling. *pnt-lacZ* is specifically expressed in PFC from stage 6 onward in the wild-type egg chamber (Fig. 2A) [39], [40]. In *hpo* mutant PFC, *pnt-lacZ* expression was disrupted in a cell-autonomous manner (96%, n = 23) (Fig. 2B) [33], [34]. This cell-fate defect was confirmed by another PFC fate marker, 667/9-lacZ (Gonzalez-Reyes, Elliot, Deng, Pathirana, Deak, Glover, St Johston, and Bownes, unpublished data) (Fig. 2D). These results suggest that Hpo signaling is required for PFC differentiation in a cell-autonomous fashion.

Disruption of any of the EGFR, JAK-STAT, or Notch signaling pathways also results in loss of expression of *pnt-lacZ* in the PFC. To determine whether the Hpo pathway regulates *pnt-lacZ* expression by affecting these signaling pathways, we applied pathway-specific markers in the mosaic egg chambers. JAK-STAT signaling is activated in a graded pattern in the FE; the highest levels are at the two termini of the egg chamber. Activation of JAK-STAT signaling can be marked by the expression of *domeless-lacZ* (*dom-lacZ*) (Fig. 2E) [30]. In *hpo* mutant clones, *dom-lacZ* was correctly expressed in the terminal follicle cells, including the PFC (Fig. 2F). In addition, we found that *slbo-lacZ*, a marker for the border cells, a group of JAK-STAT-induced anterior follicle cells [41], [42], was normal in *sav* border-cell clones (Fig. 2H). These data indicate that the activity of JAK-STAT signaling is undisturbed in follicle cells with disrupted Hpo signaling.

Specification of the PFC fate requires EGFR signaling to be activated by Grk secreted from the oocyte posterior. PFC with aberrant EGFR signaling adopt the default anterior-follicle-cell (AFC) fate, which is indicated by expression of AFC-fate markers such as *slbo-lacZ* or *dpp-lacZ* in mutant PFC [43]. In *hpo* or *sav* PFC clones, no expression of these markers was detected (Fig. 2H,J), so these cells have not taken the AFC fate. EGFR signaling is therefore unlikely to be the cause of loss of *pnt-lacZ* expression in PFC clones of the Hpo pathway mutants.

### Notch signaling is disrupted in the Hippo pathway mutants

Notch signaling, which is activated at stage 6/7, induces follicle-cell differentiation and transition from the mitotic cycle to the endocycle in the FE. Disruption of Notch signaling results in continued proliferation and expression of mitotic markers and immature cell-fate markers in follicle cells beyond stage 6 [27]. Using antibodies against mitotic markers Phospho-Histone 3 (PH3) and Cyclin B in *sav* mosaic egg chambers, we found that the PFC clones showed prolonged oscillating patterns of PH3 and Cyclin B expression after stage 6 (Fig. 3B,D). In addition, the nuclei in *sav* and *hpo* mutant PFC were much smaller than those of the wild type (Fig. 1B,C). Because extended expression of mitotic markers and smaller nuclei were also detected in *Notch* mutant follicle cell clones during midoogenesis [27], we examined the expression of several targets of Notch signaling in the mosaic egg chambers. Cut, a homeobox protein that is downregulated by Notch in the FE [44], showed continued expression in *sav* and *wts* PFC clones after stage 6 (Fig. 3F,G), whereas Hindsight (Hnt), a zinc-finger protein that is induced by Notch [45], was not expressed in the PFC clones of *hpo* or *sav* during midoogenesis (Fig. 3J,K). Because Yki is the important link between Wts and downstream transcriptional regulation of Hpo signaling [13], we examined the expression pattern of Cut and Hnt in *yki* overexpressing clones. Indeed, we found continued Cut expression (64%, n = 76) (Fig. 3H) and reduced Hnt expression (58%, n = 59) (Fig. 3M) in *yki* overexpressing clones. Although the Cut and Hnt expression defects were restricted to the mutant PFC after stages 7/8, similar defects were sometimes evident in follicle cell clones located at the anterior or lateral regions of the FE at stage 7 (Fig. 3L), suggesting the Hpo pathway can also affect Notch activity in the entire FE around stage 7.

To confirm that Notch signaling is attenuated in Hpo pathway mutants, we used E(spl)-CD2, which contains the Su(H) binding sites and the CD2 reporter, to measure Notch activity [46]. Normally, Notch activity, as indicated by CD2 staining, is upregulated in follicle cells during stages 7–10A of oogenesis (Fig. 3N). In *sav* follicle-cell clones, however, CD2 staining was significantly reduced in mutant PFC (Fig. 3O). The defects in E(spl)-CD2 expression are consistent with the aforementioned Cut upregulation and Hnt downregulation phenotypes in the FE. Taken together, our data suggest that Hpo signaling promotes Notch activation in follicle cells, particularly in the PFC.

**Figure 4:**
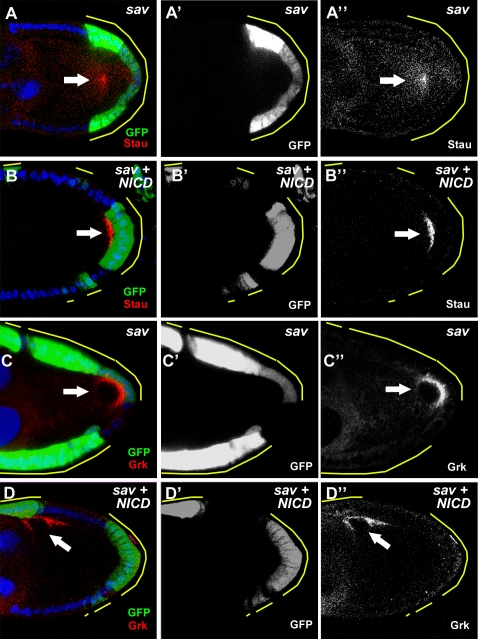
Rescue of Hpo phenotypes through overexpression of NICD. GFP-positive *sav* clones were created by the MARCM technique. (A) Stau is mislocalized to the center of the oocyte when large *sav* clones are located at the posterior. (B) Stau is localized correctly to the posterior pole when NICD is expressed in *sav* PFC clones. (C) Grk was detected at the oocyte posterior when large *sav* PFC clones were generated . (D) Misexpression of NICD in *sav* PFC clones restored Grk at the dorsal-anterior corner of the oocyte.

To determine whether the oocyte polarity defects in Hpo pathway mosaics are related to disrupted Notch signaling, we used the MARCM technique [47] to misexpress a constitutively active form of Notch, the Notch intracellular domain (NICD) [48] in *sav* mosaic egg chambers. The GFP-marked *sav* MARCM follicle-cell clones reproduced the oocyte-polarity defects, as revealed by Stau (Fig. 4A) and Grk (Fig. 4C) staining. In contrast, when NICD was expressed in *sav* PFC clones, the polarity phenotypes were rescued as these egg chambers showed significantly higher percentages of correct Stau (53%, n = 72) (Fig. 4B) and Grk/oocyte nucleus (42%, n = 65) localization (Fig. 4D); compare to *sav* clones without NICD expression: correct Stau localization (3%), correct Grk/oocyte nucleus localization (7%). These results demonstrate that the oocyte polarity defects caused by defective Hpo signaling can be attributed to disruption of Notch activation in the PFC.

### Endocytosis is defective in Hpo pathway mutant follicle cells

**Figure 5:**
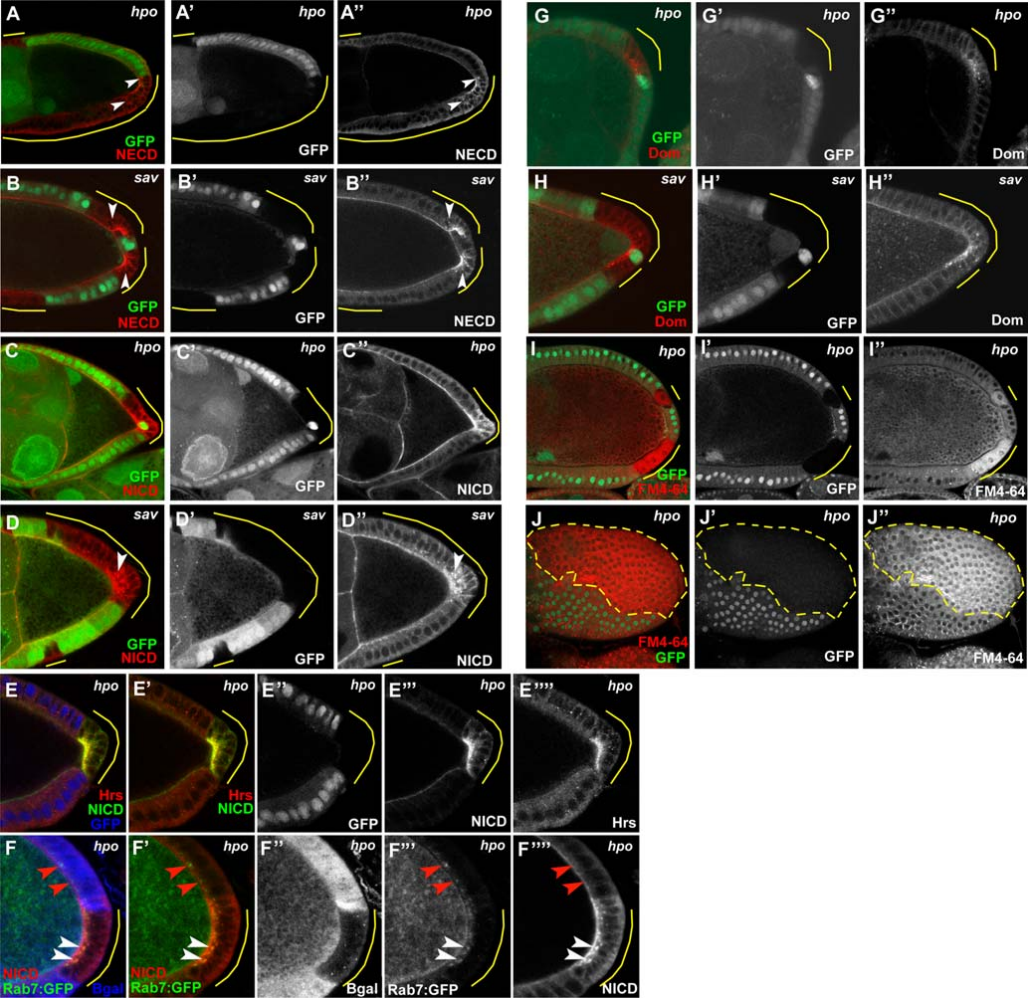
Defective endocytosis in Hpo pathway–mutant follicle cells. Both NECD and NICD accumulate in *hpo* (A,C) and *sav* (B,D) PFC clones of stage 9/10 egg chambers, including ectopic cytoplasmic puncta (arrowheads). (E) In *hpo* follicle-cell clones (indicated by loss of GFP, false-colored blue in panel E, white in E’’), Hrs (red) accumulates at the apical region and overlaps significantly with NICD (false-colored in green to faciliate determination of colocalization by yellow signal, as shown in E'). (F) Some ectopic NICD is also found to colocalize with Rab7:GFP positive vesicles (white arrowheads) in *hpo* follicle cell clones (visualized by loss of lacZ in blue in panel F, white in F’’). Note in wildtype cells the Rab7:GFP-positive vesicles do not appear to contain Notch protein (red arrowheads). *hpo* (G) and *sav* (H) mutant PFC also contain discrete cytoplasmic as well as membrane-associated accumulations of Domeless protein. Staining of the endocytic marker FM4-64FX was significantly higher in *hpo* mutant follicle cells of stage 10 egg chambers, regardless of position in the FE (I cross-section, J top view-clone outlined in dashed yellow line).

To further investigate the consequences of defective Hpo signaling on the Notch pathway, we compared the expression and localization pattern of the Notch receptor itself in wildtype cells to that of Hpo pathway mutant follicle cell clones. In *hpo* and *sav* mutant PFC, we observed significant accumulations of both NICD and the Notch Extracellular Domain (NECD), indicating that full-length Notch receptor is present in excess amounts in these clones (Fig. 5A,B,C,D). Interestingly, the ectopic Notch protein in these mutant cells was not only visible at the apical surface, as in wildtype cells, but also in punctate cytoplasmic concentrations indicating that some of the Notch protein was accumulating in discrete vesicles. This pattern suggests a potential defect in endocytic trafficking of Notch in mutants of the Hpo pathway. We therefore generated Hpo pathway follicle cell mosaics and examined the expression patterns of two key components of endocytic trafficking: Hrs (hepatocyte growth factor–regulated tyrosine kinase substrate), which is required for sorting of ubiquitinated membrane proteins to late endosomes [49], and Rab7:GFP, a marker for late endosomes [50]. In *hpo* mutant cells, we observed dramatic accumulations of Hrs, as well as significant colocalization of Hrs with NICD (Fig. 5E) in the subapical region. In addition, we found that several of the ectopic Notch-positive vesicles present in *hpo* clones were also Rab7:GFP positive (Fig. 5F), suggesting that some of the Notch protein had progressed to late endosomes.

Because Hrs is a general component of the endocytosis machinery, we tested the possibility that the higher levels of Hrs in *hpo* mutant cells might reflect differences in the overall rate of endocytosis in hpo follicle clones by incubating live mosaic egg chambers with a fluorescently-tagged, lipophilic styryl dye, FM4-64. The mosaic egg chambers were briefly incubated with the dye then allowed to internalize the dye from the plasma membrane for 30 minutes. The egg chambers were then fixed and prepared for image analysis. We found *hpo* mutant cells showed more signal than the wildtype cells, and that the staining in the clones cell displayed a diffuse cytoplasmic pattern compared to the wildtype cells which tended to be present at the membrane or in a few cytoplasmic vesicles (Fig. 5I,J). This difference was readily visible in as early as stage 7 egg chambers, but was quite pronounced by stage 9/10. These findings suggest that *hpo* mutants are more readily internalizing this dye, consistent with generally increased levels of endocytosis. In addition, we also stained *hpo* and *sav* mosaic egg chambers with Domeless antibody to see if this receptor might also be affected [51]. Similar to Notch we observed punctate accumulations of Domeless protein in the cytoplasm of PFC clones (Fig. 5G,H), whereas wildtype cells showed virtually no staining in these stages, with the exception of the polar cells which appeared to have some Domeless staining throughout midoogenesis. Taken together, our data suggest that endocytic trafficking, including endocytosis of the Notch receptor, is affected in the Hpo pathway mutants.

### Expression of the Hippo pathway targets in mutant follicle cells

**Figure 6:**
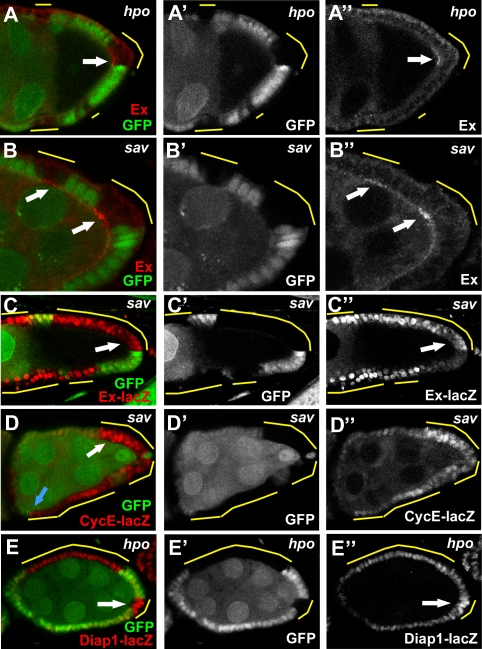
Expression of Hpo target genes in mosaic egg chambers. (A,B) Upregulated Ex expression was found in *hpo* (A) and *sav* (B) mutant follicle cells (arrows). Note that a stronger upregulation of Ex was found in the PFC clone (B). (C,D) Upregulated expression of lacZ markers for the Hpo pathway target genes *ex* (C) and *cycE* (D) was observed in *sav* follicle-cell clones (arrows). Note differences in expression of *cycE-lacZ* at boundaries between wildtype and clone cells at posterior (white arrow), as well as more anterior locations (blue arrow) in this stage 7 egg chamber. (E) In addition, *diap-lacZ* (arrow) was also upregulated in *hpo* PFC clones.

Ex expression is regulated by the Hpo pathway in a negative feedback loop. This regulation seems to be independent of cell type and tissue [15]. We found that *hpo* and *sav* mutant follicle cells had higher levels of Ex expression than neighboring wildtype cells (Fig. 6A,B), phenotypes similar to those described in the imaginal discs [15]. Interestingly, the mutant cells at the posterior had a greater upregulation of Ex expression than did the non-posterior lateral follicle-cell clones (Fig. 6B); consistent with the greater disruption of Notch signaling at the PFC. Furthermore, using *lacZ* reporters of the three negatively regulated targets of the Hpo pathway in imaginal discs, *ex*, *cycE*, and *diap1* [6], [7], [8], [15], we found upregulated expression of *ex-lacZ* and *cycE-lacZ* in *sav* follicle-cell clones (Fig. 6C,D) and upregulation of *diap1-lacZ* in *hpo* clones (Fig. 6E). These results suggest that the regulatory circuitry of the Hpo pathway in the FE is consistent with that reported in other tissues.

**Figure 7:**
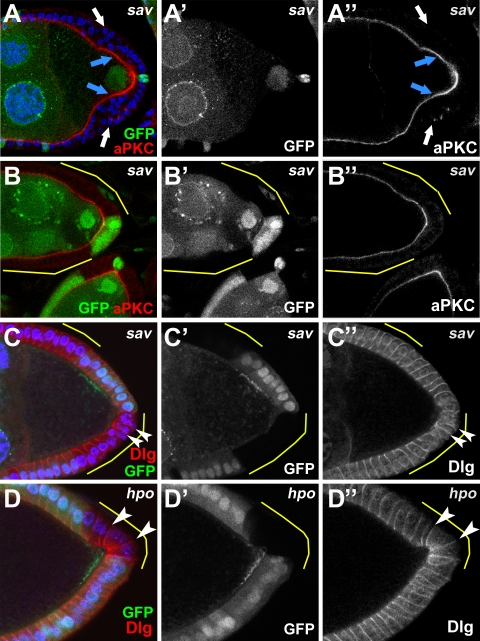
Apical-basal polarity in Hpo-defective follicle cells. *sav* mutant follicle cells that remain in contact with the germline display correct localization of the apical marker, aPKC (A, blue arrows; compare to B which shows wildtype cell pattern (GFP-positive cells) as well as no obvious defects in neighboring clone cells). *sav* clone cells in outer layers of a multilayered clone, however, do not show apical accumulations (A, white arrows). Localization of the basal-lateral marker Dlg appears largely correct in both *sav* (C) and *hpo* (D) clones (white arrowheads), but see Results for further description.

During eye development, disruption of the Hpo pathway results in an overgrowth phenotype [7], [8], [12], [15], [17], reflecting the tumor-suppressor function of the pathway. During oogenesis, when large follicle-cell clones cover the posterior half of the egg chamber, a multiple-cell-layer phenotype was frequently observed (*hpo* clones, 83%, n = 54)(Fig. 1B,C, 3G). Multilayering of the FE has been reported for mutants affecting the apicobasal polarity of the follicle cells themselves. We therefore examined the localization patterns of aPKC, an apical marker for epithelial cells [52], and Dlg, a basal-lateral marker [53] in Hpo defective follicle cells. We find that *sav* clone cells in contact with the germline maintain correct apical localization of aPKC (Fig. 7A,B), and both *sav* and *hpo* clones appear to possess correct lateral Dlg staining (Fig. 7C,D). In multilayered clone cells that have lost contact with the germline, however, we find evidence that cell polarity is disrupted because aPKC does not localize properly to the apical surface. Dlg defects are somewhat more difficult to determine in the outer cells of a multilayered overgrowth because the outer cells frequently tend to lose their columnar morphology, which in and of itself likely reflects disruption of apicobasal polarity. However, in outer cells which have lost contact with the germline yet roughly maintain a columnar appearance, Dlg appears enriched at the lateral membranes as in wildtype cells (Fig. 5C,D). Our findings are quite similar to those of Meignin et al. [34], however Polesello and Tapon report generally defective follicle cell polarity in multilayered Hpo mutant follicle cell clones [33]. Interestingly, both reports indicate that the orientation of the mitotic spindle is defective (not in parallel to the follicle cell-germline membrane connection), which has been suggested to underlie some multilayering phenotypes [54]. If follicle cell polarity truly is intact for the inner layer of cells, then it is intriguing that the mitotic spindle orientation appears to be uncoupled from these other indicators of cell polarity in these mutant cells. Nevertheless, based on the markers we have examined, we do not find significant support for a direct role of the Hpo pathway in establishing or maintaining follicle cell polarity in cells that are in contact with the germline.

### *merlin* and *expanded*, but not *fat,* have roles similar to those of *hippo*, *warts,* and *salvador* in oogenesis

**Figure 8:**
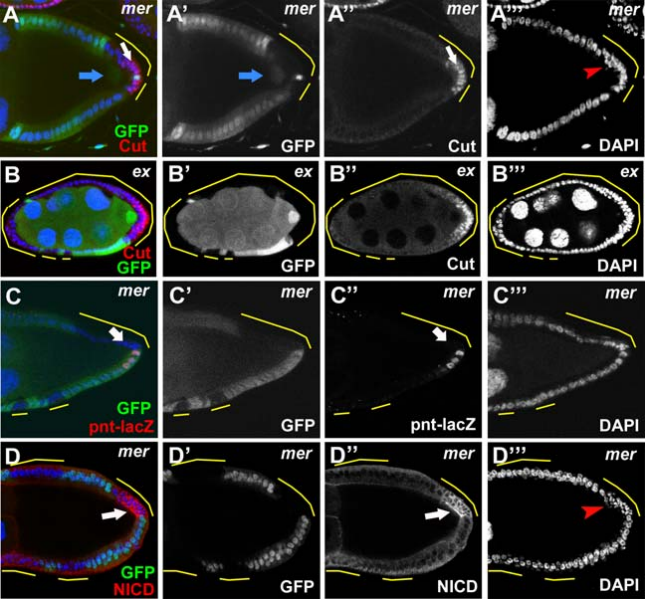
*mer* mutation disrupts PFC fate and Notch signaling. (A) *mer* clones lead oocyte nucleus mislocalization (blue arrow), and misexpression of Cut (white arrow) in the PFC clones of this stage 10 egg chamber. (B) Similar defects can also be observed in *ex* clones of this stage 7 egg chamber, although the penetrance was significantly lower (see Results). (C) Loss of *pnt-lacZ* expression was observed in *mer* PFC clones (arrow). (D) *mer* PFC clones accumulate high levels of NICD. Multilayering and small nuclei could also be seen in *mer* PFC clones (A’’’,D’’’, red arrowheads).

Previous work in imaginal discs has shown that *mer* acts upstream of *hpo* in the pathway [15]. Interestingly, a temperature-sensitive (ts) mutant of *mer* (*mer^ts1^*) causes oocyte polarity defects when raised at a restrictive temperature [55]. In contrast to our findings that the Hpo pathway is required for PFC differentiation, no obvious follicle-cell fate defects were reported in these *mer* ts mutant egg chambers. To determine whether *mer* acts independently of the core components of the pathway, we reexamined the role of *mer* in oogenesis by generating follicle-cell clones using a null allele, *mer^4^* [56]. The oocyte polarity and follicle cell multilayering defects produced in egg chambers containing *mer^4^* PFC clones (Grk/oocyte nucleus mislocalization: 87%, n = 69) were similar to those of *hpo*, *sav* and *wts* mosaics and the previously reported *mer^ts1^* phenotype (Fig. 8A, D) [55]. Also similar to other Hpo pathway mutants, PFC differentiation was defective in *mer^4^* mosaics, as indicated by loss of *pnt-lacZ* expression in PFC clones (Fig. 8C) and continued staining of Cut after stage 6 of oogenesis (Fig. 8A), suggesting that Notch activity is perturbed. In the mutant follicle cells, Notch protein accumulation was also detected (Fig. 8D), similar to our observations for *hpo* and *sav* mutants (Fig. 5A,B,C,D). Together, our results suggest that *mer*, like other Hpo pathway components, regulates Notch activity in the PFC and that its involvement in oocyte polarity formation is related to its role in follicle-cell differentiation.

*ex* has been reported to act redundantly with *mer* in the Hpo pathway in imaginal discs [15]. To determine whether mutations in *ex* would display phenotypes similar to those of *mer* in oogenesis, we performed clonal analysis using the loss-of-function allele *ex^e1^* [57]. *ex* follicle-cell clones showed oocyte polarity defects and and defects in Cut downregulation after stage 6 (Fig 8B). Compared with the core components of the Hpo pathway, however, the phenotypes of the *ex* clones were not as severe. Specifically, Cut was occassionally upregulated in the PFC of stage 7/8 ex clones (36%, n = 83), and the penetrance of oocyte polarity defects (11%, n = 54) was lower than that for *mer* and other core components of the Hpo pathway. Nonetheless, these *Notch*-like defects suggest that *ex* plays similar but possibly less essential roles in these aspects of oogenesis.

*ft* has been suggested to be the most upstream component of the Hpo pathway identified so far [17], [18], [19]. To determine whether *ft* is also required for follicle-cell differentiation and oocyte polarity, we generated follicle-cell clones of three separate alleles of ft: *ft^422^* [58], *ft^G-rv^* [59], and *ft^8^* [60], but no oocyte polarity or follicle-cell fate defects were detected (data not shown). Therefore, the Hpo pathway is probably activated in a Ft-independent manner in the follicle cells.

## Discussion

Coordinated regulation of signaling pathways is vital for proper development of multi-cellular organisms. During oogenesis, follicle-cell differentiation along the AP axis is a key step in the proper development of the egg chamber and the establishment of oocyte polarity. Here we show that the Hpo tumor-suppressor pathway joins the Notch, EGFR, and JAK-STAT pathways in regulating follicle-cell patterning and oocyte AP polarity formation. Hpo signaling promotes Notch signaling in the FE; this role is dramatically enhanced in the PFC as indicated by the restriction of any of the phenotypes we report to clones in the posterior region of the egg chamber after stage 7/8. Disruption of the Hpo pathway in the PFC results in continued proliferation and failure to differentiate, which lead to defects in AP axis formation. Alleviation of the oocyte polarity defects by expression of a constitutively active form of Notch in *sav* clones suggests that the Hpo pathway acts on these developmental processes by regulating Notch activity in these cells.

### Hippo regulates Notch receptor levels in follicle cells

Previous studies have identified several genes involved in Notch receptor trafficking and turnover in the imaginal discs [61], [62], [63], [64], [65], [66], [67], [68], [69]. Based on these studies, it appears that Notch is ubiquitinated by specific E3 ligases, thus targeting Notch for endocytosis and ultimately lysosomal degradation. The present study shows that the Hpo pathway is required to regulate Notch receptor levels in the follicle cells, and that this regulation might be achieved by promoting proper endosomal trafficking of Notch. In Hpo-pathway-mutant follicle cells, we observed a punctate distribution of Notch in the cytoplasm, as well as accumulation of the endocytic vesicle marker Hrs and its colocalization with Notch. This pattern of colocalization supports the idea that the punctate Notch staining found in Hpo mutant follicle cells reflects the accumulation of Notch in endocytic vesicles. This conclusion is bolstered by the overlap of ectopic Notch with the late endosomal marker Rab7 in Hpo pathway-defective cells. It has also been reported in *Drosophila* imaginal discs that the Hpo pathway components *ex* and *mer* regulate membrane receptor trafficking, including the Notch receptor [70]. Simultaneous loss of both *ex* and *mer* function causes accumulation of Notch at the membrane. The authors suggest that *ex* and *mer* are required for continuous clearance of the Notch receptor from the plasma membrane. Here we demonstrate that loss of *mer* function alone in PFC clones can also lead to accumulation of the Notch receptor. These findings together with our data testing core components of the Hpo pathway in the follicle cells strongly support the idea that Hpo signaling is involved in the regulation of Notch endocytic trafficking. The increased levels of Hrs and the accumulation of Domeless in cytoplasmic vesicles in PFC clones of Hpo pathway mutants suggests that the endocytosis defects we observed are probably not specific to the Notch receptor, but rather may indicate more generalized defects in endocytosis in these cells, and indeed we did observe increased staining of the non-specific endocytic marker, FM4-64 in Hpo-defective follicle cells. The fact that this marker showed a more diffuse cytoplasmic staining in the clones relative to the more punctate or membrane-associated staining observed in wildtype cells may reflect more unstable cell membranes, which could in turn facilitate uptake of the dye, thus its stronger signal in the clone cells.

Although there is a growing body of evidence indicating endocytosis and endosomal trafficking of the Notch receptor play important roles in the regulation of Notch activity [61], [62], [63], [64], [65], [66], [67], [69], this relationship is not entirely understood. Furthermore, the vast majority of the work in this area has focused on the imaginal discs, which makes any interpretation of our findings regarding Notch accumulation in Hpo mutant follicle cells particularly difficult. As an example of the complexity of this situation, mutations in many of the genes involved in Notch trafficking cause Notch accumulation and ectopic Notch activity in imaginal disc cells, whereas we find Notch accumulation and decreased Notch activity in follicle cell clones of Hpo pathway mutants. A possible explanation for this discrepancy is tissue-specific differences in the relationship between Notch trafficking defects and Notch activity. Further research in the areas of Notch trafficking and its effects on Notch activation, particularly in the follicle cells, will be very helpful in determining if the role of Hpo signaling in promoting Notch activity during oogenesis is mediated by regulation of Notch trafficking.

### Asymmetry of Hippo signaling along the AP axis in the follicular epithelium

The dramatic suppression of Notch activation in PFC clones of Hpo mutants compared to the modest and brief defects in clones present in non-posterior follicle cells is intriguing. The AP asymmetry of Notch regulation by Hpo signaling suggests the involvement of other signaling pathways that are activated in an AP gradient within the FE. The major difference between the PFC and the other cells of the FE is that EGFR signaling is exclusively activated in the PFC in response to Grk from the oocyte. EGFR activation in the PFC may repress Notch activity levels in these cells, in which case Hpo signaling might serve to antagonize this repressive function of EGFR on Notch signaling. In line with this hypothesis, MacDougall et al. reported that the multiple-cell-layer phenotype of *mer^ ts1^* was suppressed by a *grk* mutation [55]. To test the possibility that EGFR activity in the PFC augments the requirement for Hpo signaling for proper follicle-cell differentiation and thus oocyte polarity, we generated *sav* PFC clones in a *grk−/−* background to test this hypothesis further. These double-mutant egg chambers continued, however, to show defects in follicle-cell maturation and oocyte polarity, similar to the *sav* PFC clones alone (data not shown). In addition, expression of a dominant active form of EGFR, λTop [71], in *sav* follicle-cell clones located at a non-posterior region in the egg chamber did not exhibit the degree of cell-differentiation defect that was shown in *sav* PFC clones alone. These two lines of evidence argue against the hypothesis that the AP asymmetry of Notch regulation by Hpo signaling is EGFR dependent, although we cannot rule out the possibility that the stronger Notch-like defects in Hpo mutant PFC depend on the combined action of multiple signaling pathways, for example EGFR and JAK-STAT. If this is the case, the disruption of one of these pathways in the PFC would not be sufficient to suppress the Hpo mutant phenotypes, nor would ectopic activation of one pathway in non-PFC Hpo clones be sufficient to generate the phenotypes seen in the PFC clones alone.

Whether this AP asymmetry of Notch regulation is a reflection of intrinsic differences in Notch signaling levels between the PFC and other follicle cells remains unclear. Use of an antibody against the Notch ligand, Dl, to stain the egg chambers revealed that Dl expression in the oocyte is lower than that in the nurse cells during midoogenesis (data not shown). The intensity of Notch signaling in the PFC may therefore not be as strong as in other follicle cells, and may depend more on facilitators such as Hpo to achieve greater activity levels. Thus, Hpo signaling might have a general role in regulation of Notch activity in follicle cells but this regulation is more critical in a sensitized background. PFC, as well as early stage anterior and lateral follicle cells, might be such a background where the Notch activity is relatively low, therefore even minor effects could be easily detected. Alternatively, an AP asymmetry of Hpo activity might occur in the FE, consistent with our observation that the PFC clones of Hpo pathway genes showed higher levels of Ex expression than anterior or main-body clones. Presently, because the activating signal of the Hpo pathway is unknown, and because no positive targets of the pathway have been described, there is no clear test for the presence of a possible gradient of Hpo activity among the follicle cells.

### The Hippo pathway in oogenesis

*hpo*, *sav*, and *wts* mutants all show dramatic overgrowth phenotypes in eye imaginal discs [7], [8], [10], [11], [12]. They have been characterized as the core components of the Hpo pathway by means of both genetic and biochemical interactions. These genes also appear to function as core components of the Hpo pathway in the follicle cells, as evidenced by nearly identical phenotypes observed in mutant clones of these genes, including severe disruption of Notch signaling in PFC and subsequent oocyte polarity defects.

Genetic evidence suggests that *ex* and *mer* function redundantly as upstream components of the Hpo pathway [15]. Mutation of either *mer* or *ex* alone in the imaginal discs does not produce any obvious changes in phenotype. In follicle cells, however, mutation of either gene produces defects in cell differentiation. The intensity of the *mer* defects was comparable to those of the three core components of the Hpo pathway, whereas *ex* mutants displayed modest phenotypic effects. The difference between the egg chamber and the imaginal discs in the degree of *mer* and *ex* redundancy could result from increased sensitivity to genetic perturbations in the FE relative to the discs. For example, *Su(Dx)*, the negative regulator of Notch signaling, was reported to show defects in follicle cells but not in the imaginal discs [62]. Interestingly, in follicle cells *mer* produced a much stronger phenotype than *ex,* indicating that the upstream signal may act mainly through *mer* to regulate the Hpo pathway in follicle cells and that *ex* facilitates *mer* in transducing this signal.

In imaginal discs, *ft* displays a phenotype similar to that of other Hpo pathway mutants. Genetic epistasis analysis has placed it upstream of other components of the Hpo pathway in the regulation of growth and cell survival [17], [18], [19]. In follicle cells, a role for Ft in Hpo signaling is not apparent, as *ft* mutants had no oocyte-polarity or Notch-signaling defects in oogenesis, distinct from the other Hpo pathway components we investigated here. Therefore, Hpo signaling in follicle cells acts independently of Ft, suggesting that Ft is probably not a core component of the pathway. Discovering the upstream receptor for Hpo signaling in follicle cells will be of great interest.

### Hippo signaling and cell differentiation

The Hpo pathway has been shown to play critical roles in the regulation of cell proliferation, apoptosis, and growth, but little is known of the effects of this newly identified pathway on the process of cell differentiation. One recent study showed that complete loss of *ex* in eye discs had a strong inhibitory effect on photoreceptor differentiation, possibly through regulation of Wingless protein levels [72], but no visible Wingless protein abundance was found in *mer* or *hpo* mutants, and the photoreceptors differentiated normally, suggesting the *ex*-dependent regulation of photoreceptor differentiation does not require the Hpo pathway. In the present study, we observed strong PFC differentiation defects in mutants of all three Hpo pathway core components, as well as *mer*, and demonstrated that the differentiation failure in Hpo pathway mutants stems from disruption of Notch signaling. The Hpo pathway may have a conserved function in the regulation of cell differentiation through control of proper Notch activity. Investigation of the regulation of cell differentiation by the Hpo pathway in other tissues where Notch signaling is critical would be worthwhile.

## Materials and Methods

### Fly stocks

The following fly stocks were used to generate Hpo pathway mutant clones by means of the FLP/FRT system [73]: *ft^422^* [58], *ft^G-rv^* [59], *ft^8^* [60], *ex^e1^* [57], *mer^4^* [56], *hpo^42^* ^–47^ [12], *sav^shrp1^* [6], and *wts^x1^* [32]. The microtubule polarity marker *Kin:βGal;* the oocyte polarity marker *Stau-GFP*; the AFC markers *slbo-lacZ* and *dpp-lacZ;* the PFC markers *pointed-lacZ* and 667/9 line; the JAK/STAT pathway–specific marker *Domeless-lacZ;* the Hpo pathway target gene reporters *ex-lacZ*, *diap1-lacZ*, and *cycE-lacZ;* the trafficking marker *Rab7-GFP* [50]; and the Notch activity reporter *E(spl)mβ-*CD2 were incorporated into corresponding Hpo pathway mutant clone backgrounds. For rescue analysis, the following stocks were used: UAS-NICD [an active form of Notch [74]], UAS-λTop [71]. Flies with Grk; sav double-mutant clones had the following genotype: hsflp; *grk^2B6^*/*grk^HK^*; FRT82B *sav^shrp1^*/FRT82B GFP.

### Clone Generation and Immunohistochemistry

Follicle cell clones were generated by 37°C heat shock of second- and third-instar larvae for 2 h, except for *wts^x1^* clones which were generated by 37°C heat shock of adult flies twice daily for 1h. All flies were put in fresh food vials with wet yeast for 3–4 days before dissection.

Antibody stainings were carried out according to a standard antibody staining protocol. The following antibodies were used: mouse anti-Cut, 1∶50; mouse anti-Dlg, 1∶20; mouse anti-Grk, 1: 40; mouse anti-Hnt, 1∶15; mouse anti-Notch, 1∶15 (NICD and NECD); mouse anti-CycB, 1∶50 (Developmental studies Hybridoma Bank (DSHB)); mouse anti-CD2, 1∶50 (ABD Serotec); anti-Domeless, 1∶200 [51]; guinea-pig anti-Ex, 1∶3000 (a gift from R. Fehon); rabbit anti-β-Galactosidase, 1∶5000 (Sigma); guinea-pig anti-Hrs, 1∶1000 (Lloyd et al. 2002); rabbit anti-PH3, 1∶200 (Upstate Biotechnology); rabbit anti-aPKC, 1∶1000 (Santa Cruz Biotechnology); Rabbit anti-Stau, 1: 1000 (gifts from D. St Johnston and P. MacDonald).

The endocytosis assay using the FM4-64FX (Molecular Probes) fixable dye was performed as follows. Ovaries were dissected in Schneider's medium and transferred to 10 µM solution of the dye diluted in medium. Incubation for 5 minutes was followed by three washes in medium alone, letting sit 10 minutes between each wash. The ovaries were then fixed for 15 minutes, washed in PBS twice, and mounted.
